# N-linked glycosylation sites with low occupancy support sustained circulation of the A(H3N2) influenza A virus in the human population

**DOI:** 10.1128/jvi.00662-26

**Published:** 2026-08-28

**Authors:** Irina V. Alymova, Betlehem Mekonnen, Dongxia Wang, Ram P. Kamal, Wen-Pin Tzeng, Alexander S. Jureka, John R. Barr, Shane Gansebom, Ian A. York

**Affiliations:** 1 Immunology and Pathogenesis Branch, Influenza Division, National Center for Immunization & Respiratory Diseases, Centers for Disease Control & Prevention 209773, Atlanta, Georgia, USA; 2 Division of Laboratory Sciences, National Center for Environmental Health, Centers for Disease Control & Prevention 314424, Atlanta, Georgia, USA; 3 Oak Ridge Institute for Science and Education 17215, Oak Ridge, Tennessee, USA; 4 Federal Civilian Division, General Dynamics Information Technology, Atlanta, Georgia, USA; 5 Chippewa Government Solutions, LLC, Sault Sainte Marie, Michigan, USA; Emory University School of Medicine, Atlanta, Georgia, USA

**Keywords:** influenza, viral evolution, hemagglutinin, immune evasion, glycosylation

## Abstract

Glycosylation of the influenza A virus hemagglutinin is crucial for viral fitness and immune evasion. The hemagglutinin of contemporary human A(H3N2) influenza A viruses is extensively glycosylated with up to 13 putative glycosylation sites. Glycan types and occupancy of these sites are not uniform: while most are nearly completely occupied by glycans, some, such as those at amino acid residues 45 and 144, are only partially occupied and are evolutionarily unstable. While the effects of highly occupied glycosylated sites of hemagglutinin on A(H3N2) influenza A virus biology are well studied, there is limited information on the functional impact of the low-occupancy glycosylation sites on the viral hemagglutinin. Here, by using reverse-genetics A(H3N2) influenza A viruses lacking glycans at either residue 45 or 144, or both in hemagglutinin, we demonstrate that, despite reduced receptor binding, thermal stability, and fusion, the presence of a low level of glycosylation at these positions does not impact virus growth in Madin-Darby canine kidney epithelial cell culture. In contrast, these low-occupancy sites do affect immune responses in mice, by reducing titers of antibodies that correlate with virus neutralization and protection against influenza disease. We suggest that the temporary introduction of sites with low glycosylation allows influenza viruses to reduce the immune pressure on antigenic and receptor binding sites of hemagglutinin without significantly compromising viral virulence. This mechanism may help support the sustained circulation of A(H3N2) influenza A virus in human populations.

**IMPORTANCE:**

Many of the glycosylation sites on the hemagglutinin of influenza A viruses are near antigenic sites, such that the glycans block antibody recognition and help evade population immunity. However, glycans may also reduce viral replication and virulence. Glycosylation sites at amino acid residues 45 and 144 of the hemagglutinin of contemporary A(H3N2) influenza A viruses are minimally occupied, with fewer than 10% of the sites containing glycans. This is still sufficient to partially evade antibody-based population immunity, while minimizing the impact on replication and virulence. Over time, A(H3N2) viruses have used both these sites only during limited periods, suggesting that even a low level of glycan occupancy may confer some negative selection pressure that is compensated by evasion of human population immunity. Data from this study provide a better understanding of the mechanisms that allow viruses to evade antibody-based immunity and maintain circulation in the human population.

## INTRODUCTION

Influenza A virus (IAV) of the A(H3N2) subtype has been endemic in the human population since 1968, leading to widespread population immunity (1, 2). This population immune pressure, particularly through antibodies, promotes evolutionary changes in the hemagglutinin (HA) of A(H3N2) IAV (3). HA is a trimeric glycoprotein present on the virion surface as well as on infected cells at later stages of virus replication, and is required for virus attachment to its sialic acid (SA) cellular receptors and for fusion of viral and cellular membranes during the initiation of infection (4). It is exceptionally tolerant of mutations, allowing the protein to accumulate sequence changes at sites targeted by antibodies, so that every few years, new A(H3N2) antigenic drift variants arise that escape pre-existing immunity (3, 5, 6).

**Fig 1:**
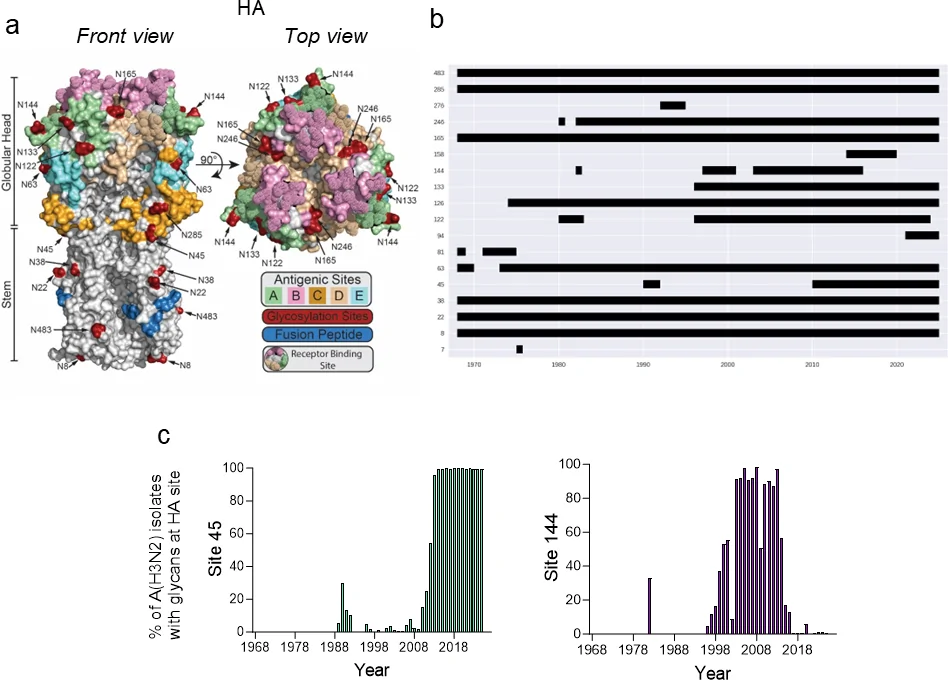
N-linked glycosylation of A(H3N2) IAV hemagglutinin. (**a**) Front and top surface views of the full-length HA H3 protein from A/Switzerland/9715293/2013 (PDB ID:6PDX) showing the glycosylation sites, antigenic sites, and fusion peptide as labeled (15). Dotted spheres represent the receptor binding site. Sites of potential N-linked glycan addition were identified based on sequence Asn-(X≠Pro)-Ser/Thr-(Y≠Pro) (where X and Y represent any amino acid except proline). Numbers indicate the start of an N*-*linked glycosylation sequon (Asn) (numbering based on the mature HA molecule after cleavage of the 16-amino-acid signal but ignoring the HA1-HA2 cleavage site; residue 483 is HA2 154 in PDB 4FNK) (16). (**b and c**) Evolutionary dynamics of N-linked glycosylation of A(H3N2) IAV HA from 1968 to 2025. Hemagglutinin protein sequences from human A(H3N2) isolates were downloaded from GISAID on 11 June 2025, manually curated to remove duplicates and low-quality sequences, and aligned using MAFFT. The year of isolation was identified from the virus description. (**b**) Frequency of NLG site at a threshold of 10.0% is shown.

Changes to the HA of 2013 A(H3N2) IAVs included amino acid (aa) substitutions that modified glycosylation of the protein. Glycosylation is a key factor in IAV pathogenicity, antigenicity, and epidemiology (7, 8). The HA of contemporary human A(H3N2) IAV strains is covered, on average, by 12 glycosylation sites; a variable number of these are on the head domain, whereas glycosylation sites in the stalk domain are relatively conserved across HA groups (Fig. 1a). Glycans that are proximal to the receptor binding site (RBS) of the globular head or the fusion (F) peptide of the stalk domain of HA can interfere with viral entry and membrane fusion (9–12). Glycans near or within antigenic sites A through E can mask these sites and reduce recognition of viruses by neutralizing antibodies (13, 14).

The presence of the consensus sequence Asn-(X≠Pro)-Ser/Thr-(Y≠Pro) (where X and Y represent any aa except proline) is required for N-linked glycosylation (NLG) of IAV HA, but the occupation of a potential site by glycans is not mandatory (17–19). Glycosylation site occupancy describes the proportion of protein molecules in a sample that are glycosylated at a specific site.

In mammalian cells, the oligosaccharyltransferase complex of the endoplasmic reticulum is responsible for the extent of NLG (20). The oligosaccharides of the glycoproteins may end in mannose (high-mannose glycans), galactose and/or N-acetylglycosamine/fucose (complex glycans), or a combination thereof (hybrid glycans) (18). Glycan composition, linkage, and structure at a specific site are affected by the activity of the glycosidases and mannosidases of the Golgi complex, among other factors.

Most studies focusing on the role of glycosylation in the biology of IAV have not directly measured NLG site occupancy and glycan composition. However, recent studies involving mass spectrometry (MS) indicate that at least 2 of the 12 NLG sites of A/Switzerland/9715293/2013 (H3N2) (“A[H3N2]2013”) IAV HA (sites N45 and N144; the numbering of NLG sites is based on the mature HA molecule without the 16-amino-acid signal sequence) are occupied by glycans only at low levels (21–23).

NLG site N45 is located within antigenic site C and is relatively distant from both the RBS and the F peptide of HA (Fig. 1a; Fig. S1) (24, 25). NLG site N144 is located within antigenic site A and is physically close to several key features of the RBS responsible for receptor specificity and binding (measuring straight-line distance between residue C-alphas in PDB:6PDX: 130-loop: ~10 Å, 190-loop: ~25 Å, 220-loop: ~19 Å; Fig. S1) (15).

Neither site 45 nor 144 comprised an NLG motif in the avian-origin HA that sparked the 1968 IAV pandemic (Fig. 1b; Table S1). Site 144 gained an NLG motif in the mid-1990s until around 2016; site N45 was briefly glycosylated in the late 1980s and early 1990s, and then again starting around 2006 (Fig. 1b and c; Table S1). In 2024–2025, less than 1% of human A(H3N2) viruses whose HA sequences are available from Global Initiative on Sharing All Influenza Data (GISAID) are glycosylated at site N144, while more than 99% contain an NLG addition site at position 45 (Table S1). During 2013, both sites were glycosylated in 94.4% of circulating A(H3N2) IAV. From 2014 to 2016, a sharp decline in circulation of viruses with two sites (N45 and N144) occurred, with fewer than 1% of human H3N2 isolates containing both sites in 2017 and later.

The location of N45 and N144 within important antigenic and functional regions of HA, their fluctuating evolutionary pattern, and their low glycan occupancy raise questions about the impact of low-occupancy glycosylated sites on IAV biology, host immune responses, and the dynamics of virus evolution.

Phylogenetic analysis and observational clinical data have suggested that sites N45 and N144 may be important for A(H3N2) IAV virulence (26, 27). The effect on antigenicity of both NLG sites has also been assessed, with the presence of N144 (but not N45) on H3 HA significantly affecting antibody-mediated neutralization, although site occupancy was not evaluated in those studies (28).

Here, we investigate the effects of low glycan occupancy at sites N45 and N144 of HA on A(H3N2)2013 IAV virulence and generation of adaptive immune responses against the virus, and suggest an important role for these sites in the continuing evolution of A(H3N2) in the human population.

## RESULTS

### Sites N45 and N144 of A(H3N2)2013 IAV hemagglutinin have low glycan occupancy

Our recent MS study with reverse genetics (rg) A(H3N2)2013 IAV (double-glycosylated rg+45/+144 virus) showed that sites N45 and N144 of HA have low glycan occupancy (23). To understand the effect of reduced glycosylation at these positions on the biology of A(H3N2) IAV, we also generated single-deletion rg−45/+144 or rg+45/−144, or double-deletion rg−45/−144, and confirmed the glycosylation pattern of their HAs in egg-grown concentrated purified inactivated stocks of each virus by MS.

**Fig 2:**
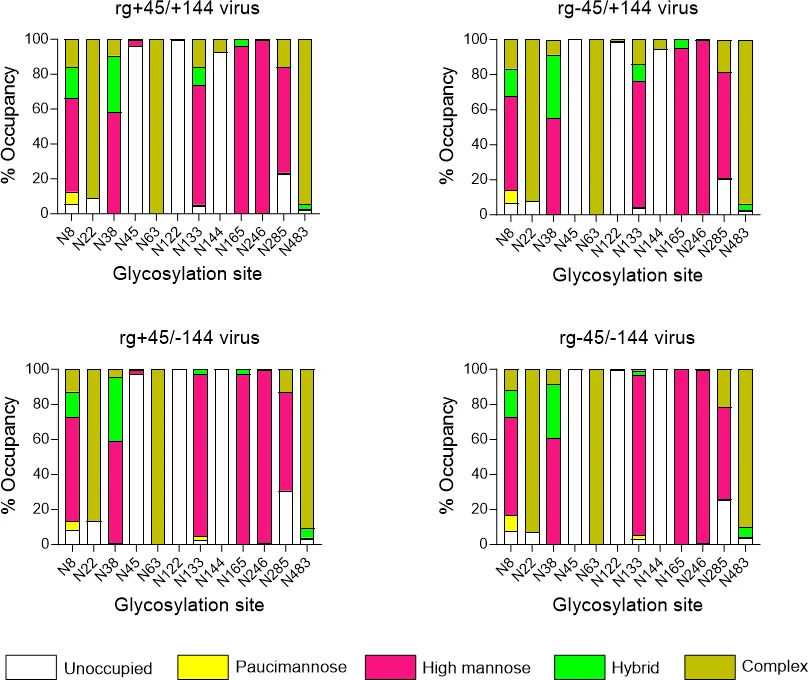
Occupancy of the NLG sites of the hemagglutinin of A(H3N2)2013 IAV. The HA proteins from four tested glycosylation mutant viruses were proteolytically digested and analyzed by nLC-MS to determine site occupancy and composition of precise glycoforms at each site. Categories of glycans are color-coded. Detailed data on glycan isoforms for each site are shown in Table S1. Glycan types include paucimannose (HexNAc_1-2_Hex_0-4_), high mannose (HexNAc_2_ Hex_5-12_Fuc_0-1_NeuAc_0-4_), hybrid (HexNAc_3_Hex_3-6_Fuc_0-1_NeuAc_0-4_), complex (HexNAc_4-9_Hex_3-10_Fuc_0-1_NeuAc_0-4_), and unoccupied.

When present, most glycan sites (except N122) were occupied by glycans, and glycan occupancy and composition of sites were generally similar in all four HA proteins (Fig. 2; Table S2). Confirming our previous data, occupancy of sites N45 and N144 was low (less than 10%) (23). Sites N45, N165, and N246 were predominantly occupied by high mannose glycans; sites N22, N63, N144, and N483 by complex glycans; site N285 by high mannose and complex glycans; and sites N8, N38, and N133 had a combination of high mannose, hybrid, and complex glycans.

### Low-occupancy glycosylation at sites N45 and N144 of hemagglutinin reduces A(H3N2)2013 IAV binding to red blood cells and thermal stability

Influenza virus infection is initiated when the RBS of the HA binds to specific α2,3- or α2,6-linked SA-containing receptors on epithelial cells of the human respiratory tract (29). To assess receptor binding of rgA(H3N2) 2013 IAVs, we normalized the amount of concentrated purified virus in the sample by determining densities of the HA bands in sodium dodecyl sulfate–polyacrylamide gel electrophoresis (SDS-PAGE) to contain approximately 0.03 μg of the HA in tested volume (50 μL) and performed hemagglutination assays. In these assays, HA binds to SA-containing receptors (regardless of the SA) on red blood cells (RBC), leading to their agglutination (30).

**Fig 3:**
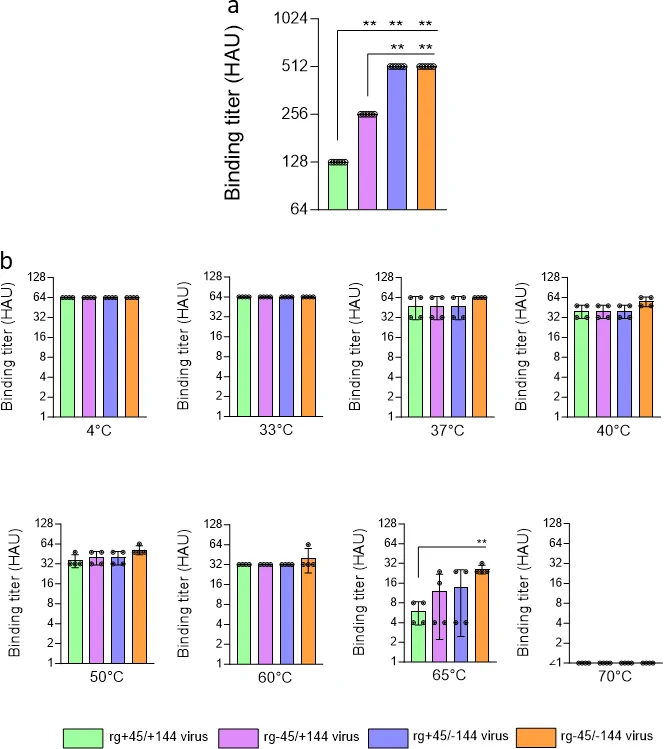
Binding of rgA(H3N2)2013 glycosylation mutant IAV to red blood cells. In binding assays, concentrated purified double-glycosylated rg+45/+144 and its glycosylation mutant virus variants (rg−45/+144, rg+45/−144, and rg−45/−144) were diluted in PBS (**a**) to provide an equal amount of HA (0.03 μg HA) in the tested sample or (**b**) to 64 HAU/50 µL. (**b**) Prior to binding assays, viruses were kept at temperatures ranging from 4°C to 70°C for 20 min. (**a and b**) Binding intensities (shown in HAU) of the viruses were recorded after incubation with 0.5% turkey RBC for 1 h at 4°C. Values from two independent experiments are shown. Error bars indicate the standard deviations (SD) of the mean. An asterisk indicates a significant difference: *P < 0.05, **P < 0.005 (**a**) by Student’s *t*-test or (**b**) one-way ANOVA with Sidak’s multiple comparison test.

The double-glycosylated rg+45/+144 had a significantly lower binding titer than any glycosylation mutant virus (*P* < 0.05) (Fig. 3a). Binding of the single-deletion rg−45/+144 was also reduced when compared with that of rg+45/−144 or rg−45/−144 (*P* < 0.05).

Glycan modifications can change the conformation and, therefore, stability of HA (31). We compared the thermal stability of rgA(H3N2)2013 IAVs HAs at 64 HAU after incubation at different temperatures in hemagglutination assays. The double-glycosylated rg+45/+144, but not the single-glycosylated variants, was less stable at 65°C than the double-deleted rg−45/−144 (*P* < 0.05) (Fig. 3b).

Therefore, low-occupancy glycosylation at either site N45 or N144 reduces the ability of A(H3N2)2013 IAV HA to bind to its receptor, and glycosylation at both sites reduces the thermal stability of HA.

### The low level of glycosylation present at site N144 of hemagglutinin is sufficient to alter the pH of membrane fusion of A(H3N2)2013 IAV

After binding SA, IAV is internalized and, in the acidic conditions of endosomes, HA undergoes a conformation change that induces fusion of the viral and endosomal membranes, allowing release of the viral genome into the cytoplasm (32). We compared the fusion of concentrated purified rgA(H3N2)2013 IAVs in a hemolysis assay (33). In these assays, after the binding of HA to the surface of RBC, we acidified the suspension of virus and RBC with sodium acetate buffer (pH ranging from 4.6 to 5.2) to trigger conformational changes in the HA associated with membrane fusion, leading to RBC rupture and release of hemoglobin (34).

**Fig 4:**
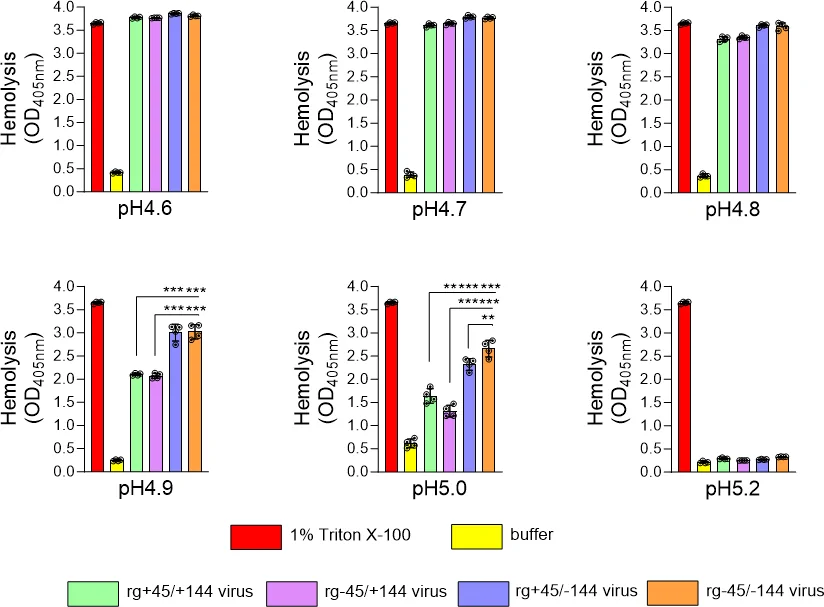
Fusion activities of rgA(H3N2)2013 glycosylation mutant viruses. In hemolysis assays, viruses were diluted in PBS to 2000 HAU/50 µL, incubated with an equal volume of 2.0% turkey RBC, and then acidified to the pH ranging from 4.6 to 5.2. The negative control was sodium acetate buffer of appropriate pH, and a solution of 1% Triton-X 100 in PBS was used as a positive control. Non-lysed RBC were removed by low-speed centrifugation, and the degree of hemolysis was determined by measuring the OD of the sample at 405 nm. Values from one typical experiment (out of a total of four) are shown. Error bars indicate the SD of the mean from four replicates. An asterisk indicates a significant difference: *P < 0.05, **P < 0.005, and ***P < 0.0005 determined using one-way ANOVA followed by Sidak’s multiple comparison test.

A(H3N2)2013 IAV containing HA with N144 (double-glycosylated rg+45/+144 and single-deleted rg−45/+144) showed reduced fusion activity at pH 4.9 and pH 5.0, compared with the viruses lacking glycosylation at this site (*P* < 0.05) (Fig. 4). Glycosylation at site N45 of HA had a negligible effect on the pH of fusion. Therefore, even the low glycosylation occupancy of site N144 is sufficient to alter the pH of fusion of A(H3N2)2013 IAV.

### Glycosylation of site N45 or N144 of hemagglutinin does not limit replication of A(H3N2)2013 IAV in Madin-Darby canine kidney (MDCK) epithelial cells

**Fig 5:**
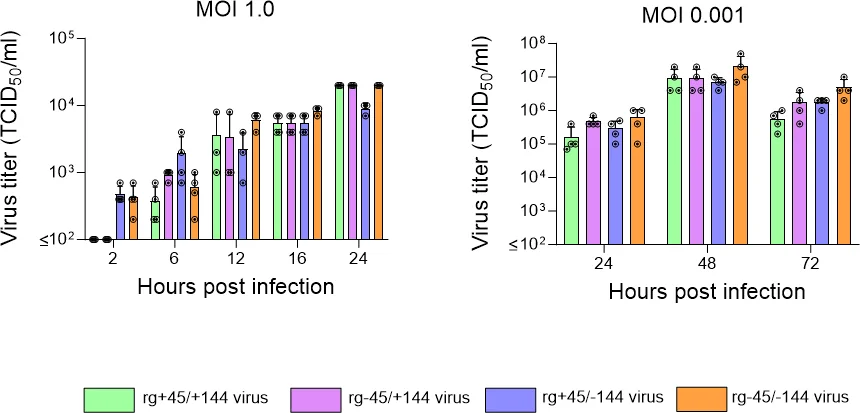
Growth kinetics of rgA(H3N2)2013 IAV in epithelial cells. Monolayers of MDCK cells were infected with rg+45/+144, rg−45/+144, rg+45/−144, or rg−45/−144 at MOI 1.0 or 0.001 at room temperature. Virus titers were determined at the times indicated on the *x*-axis (detection limit is 10^2^ TCID_50_/mL). Error bars indicate the SD of the mean of the results from four biological replicates in one representative experiment (of three total). Differences between tested viruses were not significant at *P* < 0.05 by one-way ANOVA with Sidak’s multiple comparison test. MOI – multiplicity of infection.

The reduced receptor binding associated with glycosylation of either site N45 or N144 (Fig. 3a), and the restricted fusion associated with glycosylation of site 144 (Fig. 4), could affect virus replication. We compared the replication of rgA(H3N2)2013 IAVs in MDCK cells, a cell line commonly used for influenza virus infection, at low (0.001) or high (1.0) multiplicities of infection (MOI) and at various times post-infection. No significant differences in replication were seen between the four viruses under any of the tested conditions and time points (Fig. 5).

### Interaction of A(H3N2)2013 IAV with broadly neutralizing antibodies is not altered by low-occupancy glycosylation at site N45 or N144 of hemagglutinin

Glycans can shield HA from antibody binding, reducing the effectiveness of host immunity and permitting viral replication and transmission in infected people (13, 35). We next assessed the ability of broadly cross-reactive anti-globular head and anti-stalk HA monoclonal antibodies (mAb) to bind the HA of rgA(H3N2)2013 IAVs. These antibodies target either the stalk region of all 16 subtypes of IAV (FI6); the stalk region of H3 and H7 IAV (C66); or the globular head of H1 and H3 IAV at the trimer interface (C6) (36). C9 mAb, which targets the stalk region of H1 and H5 IAV but does not bind to H3, was used as a negative control.

In enzyme-linked immunosorbent (ELISA) assay, we found no difference in the levels of binding of tested FI6 or C66 or C6 mAb between four rgA(H3N2)2013 IAV (Fig. S2). As expected, C9 mAb did not bind the HA of any tested virus.

Thus, the low level of glycosylation at site N45 or N144 does not prevent HA from interacting with broadly active mAb, suggesting that antibodies are able to access these sites equally well.

### Low-occupancy glycosylation at sites N45 and N144 of hemagglutinin reduces the immunogenicity of A(H3N2)2013 HA

To further test if the low level of glycan occupancy at sites N45 and N144 of A(H3N2)2013 IAV HA is sufficient to affect antibody responses, we immunized naïve mice with two intramuscular injections of 10 µg of HA from concentrated purified double-glycosylated rg+45/+144, single-deleted rg−45/+144 or rg +45/−144, or double-deleted rg−45/−144. ELISA, hemagglutination inhibition (HI), and neutralization assays (which measure either all antibodies that bind to the virus, or protective, or neutralizing antibodies, respectively) were used to assess serological responses against homologous and heterologous rgA(H3N2)2013 IAV at various times after immunization.

**Fig 6:**
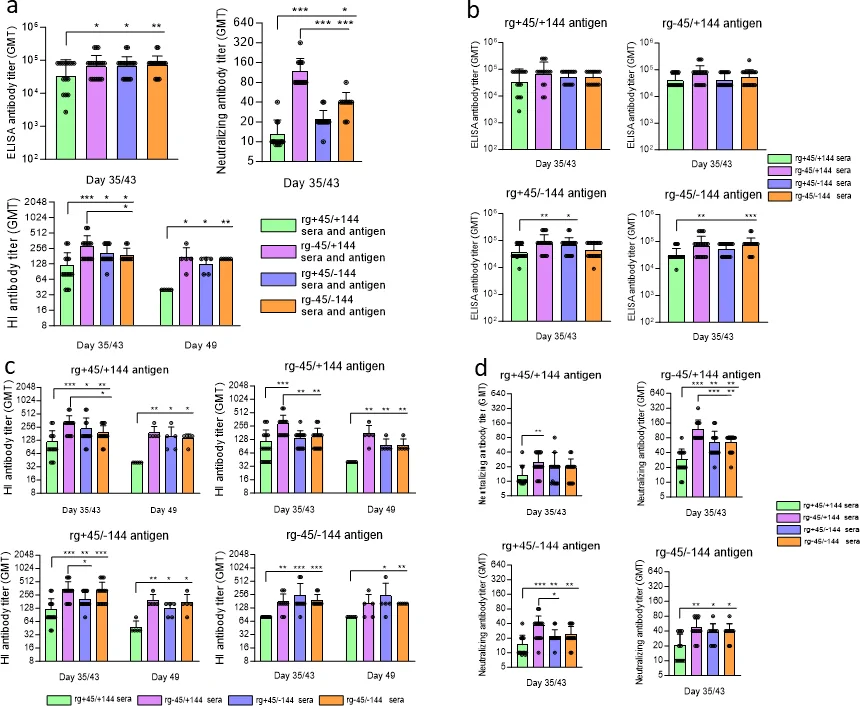
Immunogenicity of rgA(H3N2)2013 IAV in mice. (**a–d**) Mice were immunized with 10.0 µg of HA from A(H3N2)2013 IAV rg+45/+144, rg−45/+144, rg+45/−144, or rg−45/−144. Pooled antibody titers from days 35/43 (*n* = 9−20), and antibody titers for day 49 (*n* = 5) determined using homologous and heterologous virus antigens are shown. (**a and b**) Total homologous and cross-reactive antibody titers measured by ELISA. Homologous and cross-reactive HA antibody titers determined by (**a and c**) HI and (**a and d**) neutralization assays. Data represent the average (±SD) of one independent assay (out of two) run in duplicate. Significant differences between groups: *P < 0.05, **P < 0.005, and ***P < 0.0005 were determined using one-way ANOVA followed by Sidak’s multiple comparison test.

When comparing homologous antibody responses, the double-glycosylated rg+45/+144 virus was consistently less immunogenic than the glycan-deletion variants in all ELISA, HI, and neutralization tests (Fig. 6a). Single-deleted rg−45/+144 had the highest homologous titers in HI (day 35/43) and neutralizing assays.

Similar trends were observed when comparing activity of each antigen against homologous and heterologous sera titers. Serum raised against double-glycosylated rg+45/+144 virus was less reactive against heterologous rg+45/−144 and rg−45/−144 antigens in ELISA tests and against homologous rg+45/+144 and any tested heterologous antigen in HI and neutralization assays comparing the activity of sera raised against any glycosylation mutant virus (*P* < 0.05) (Fig. 6b through d). When comparing activity of the most immunologically relevant neutralizing antibodies against the different viruses, again rg+45/+144 virus was less effectively inhibited by the various sera (*P* < 0.05) (Fig. S3a through c). These observations indicate that even the low level of glycosylation at positions 45 and 144 of HA reduces homologous and heterologous serum antibody responses, particularly antibodies that correlate with protection against influenza disease and virus neutralization.

## DISCUSSION

Despite decades of A(H3N2) IAV history with humans, we still do not fully understand the mechanisms contributing to its uninterrupted circulation in the human population. It is believed that the extensive glycosylation of HA of contemporary A(H3N2) IAV shields the protein from antibodies, contributing to antigenic escape and viral virulence (13, 14, 35). However, glycan shields may also affect essential functions of HA. It has been proposed that there is a limit on the number of NLG sites that can be accommodated by H3 HA, with seven or eight sites in the globular head and five in the stalk region (Fig. 1a) (37). Our data suggest that under-occupancy of sites N45 and N144 may allow HA to tolerate this large number of glycans.

Sites N45 and N144 of A(H3N2)2013 IAV have markedly reduced levels of glycosylation compared with most other sites of HA (<10% vs nearly 100%) (Table S2). Both sites N45 and N144 contain Ser at the +3 position of their sequon, which reduces the probability of glycosylation by 2–3-fold compared with the presence of Thr at +3 (19). Almost all (>99.8%) A(H3N2) IAV with N45 and N144 sites on HA present in GISAID also have Ser at this position. Although the sensitivity of current techniques does not allow analysis of glycans on IAV from natural human infections, this observation suggests low-occupancy glycosylation of both sites in viruses circulating in humans (Table S1).

Physical proximity to another NLG site can also prevent access by glycosylation enzymes, reducing glycan occupancy of a sequon (38, 39). A straight-line distance between the two alpha-carbons of sites N144 and N63 is within ~21 Å (when measured on PyMol structure of the H3 HA in PDB:6PDX) and within ~13 Å of N133, and site N45 is within ~11 Å of N285 (Fig. S1) (15). Thus, full glycosylation at these surrounding sites could contribute to the partial occupancy of N45 and N144. Of note, the near-total absence of glycosylation at site N122 in the current study might also be a result of the presence of the glycosylation-limiting sequon (Asn-Glu-Ser) at that site as well as proximity to the fully glycosylated sites N63 (~25 Å) and N133 (~14 Å) (Fig. 2; Fig. S1; Table S2).

Sites N45 and N144 showed low glycan occupancy whether the viruses were grown in eggs (Fig. 2; Table S2) or cells (22, 23). While intrinsic sequence effects lead to low occupancy, the precise level of glycosylation may vary depending on the host, and these variations may have functional significance. This highlights the importance of measuring, rather than inferring, glycosylation when evaluating the role of glycosylation in IAV biology.

We show that even a low level of glycosylation may influence the functions and antigenicity of HA when NLG sites are situated within or close to biologically important regions. Binding to RBC was modestly reduced when glycosylation was present at site N45 and more substantially reduced when it was present on N144, even though both have low occupancy (Fig. 3a). Because site N144 is close to the RBS (especially the 130-loop: ~10 Å), glycans may shield the RBS and interfere with HA receptor binding (Fig. S1). Since site N45 is distant from the RBS (>60 Å), direct interference of glycans with the RBS does not seem likely. However, the absence of glycans at residue 45 could destabilize the whole glycan shield of HA, such that the RBS is less covered. Also, the change from Asn to Ser at +1 position of the sequon in the single-deleted rg−45/+144 virus could affect existing hydrogen bonds because of the lack of an amide group, thereby altering the conformation and biological function of the protein (40). The change in binding seen with rg−45/+144 is small, consistent with indirect effects or minor conformation changes.

The other important biological change observed in the presence of both N45 and N144 (double-glycosylated rg+45/+144 virus) was reduced thermal stability of HA (Fig. 3b). While this was detected only at nonphysiological conditions (65°C), it still indicates the potential of HA being inactivated at lower temperatures, making it ineffective in the upper respiratory tract.

The HA of current human-adapted A(H1N1)pdm09, A(H2N2), and A(H3N2) IAV have fusion activation pH values of 5.0–5.4 (32, 41). Glycosylation at site N45 did not affect A(H3N2)2013 IAV fusion with RBC at tested pHs (Fig. 4), while the presence of glycans at site N144 reduced fusion of these viruses with RBC at pH 4.9 and pH 5.0 compared with viruses without this site. Although site N144 is distant from the fusion peptide, fusion potential may be affected by receptor binding (42–45). The stronger binding observed with mutants lacking an NLG site at residue 144 could lead to more effective fusion. In addition, low pH-driven conversion of the HA trimer from its pre-fusion to post-fusion state is triggered by the protonation of histidine residues (46). Thus, another possible reason for the effect of N144 glycosylation on fusion could be that glycans at this site alter the chemical environment of nearby histidines at 156, 183, or 184, affecting their protonation.

Despite changes in receptor binding, thermal stability, and fusion observed with the glycosylation variants compared with double-glycosylated rg+45/+144 virus, the growth of the variants in MDCK cells was unaffected (Fig. 5). These data are consistent with our previous conclusion that there is a functional threshold affinity level above which infection and intercellular fusion proceed with equal efficiency so that glycosylation changes in the HA can modulate receptor binding without affecting virus virulence (44, 47).

Mice immunized with rgA(H3N2)2013 IAV containing glycosylation at N45 and N144 showed both reduced immunogenicity (i.e., the ability to induce a protective immune response in the host) and reduced antigenicity (the ability of pre-existing antibody to inhibit virus), compared with the rg virus variants lacking these glycosylation sites (Fig. 6; Fig. S3). Probably because of reduced exposure of immunodominant antigenic sites necessary for robust antibody generation (48, 49).

The effects of sites N45 and N144 of the HA on A(H3N2)2013 IAV biology are surprisingly significant considering their low occupancy. While we cannot provide a definitive explanation for these effects, we note that HA tends to form higher-order “trimers-of-trimers” clusters, especially during receptor binding (50). Those interactions between HA trimers may be influenced by their glycans (31). Assuming random interactions, each trimer-of-trimers would have about a 65% chance of having at least one protein glycosylated at one of these sites. Since multiple interactions are required for virus binding and entry and development of host immune responses, there are clearly opportunities for these low-occupancy sites to influence the biology of viruses. The biological effects of glycans also depend on their spatial orientation (51). In fully occupied sites, only some glycans can be oriented toward the receptor binding or antigenic sites. In sites with low occupancy, all attached glycans can be properly directed.

The amino acid change at the NLG site also can mediate some of the observed effects. Our immunological findings with site N144 are similar (but not identical) to those of others (28). Both research groups concluded that glycosylation at this site affects antibody responses. The different approaches used to eliminate the NLG site by us and other researchers suggest that glycosylation itself, rather than amino acid substitutions, is responsible for the observed biological effects, at least with site N144.

Our data suggest that A(H3N2) IAV uses sites with low glycan occupancy to reduce immune pressure from the host on antigenic sites and the receptor-binding site of HA without compromising viral replication. We propose that A(H3N2) IAV compensates for its numerous NLG addition sites by forcing some to only have low glycan occupancy, and by alternating the use of low-occupancy sites over time. We find that even the low glycan occupancy on such sites is sufficient to reduce viral immunogenicity. While the presence of these sites does not interfere with replication of virus *in vitro*, it does impact some biological functions of HA (such as receptor binding, thermal stability, and pH of fusion), and the fact that sites N45 and N144 rarely co-exist suggests that it may cause some negative effects on viral replication or transmission in a natural setting; thus, as population immune pressures ease, virus strains lacking one of these sites become more prevalent. We conclude that HA sites with low glycan occupancy may support sustained circulation of A(H3N2) IAV in human populations.

## MATERIALS AND METHODS

### Cell culture

The epithelial MDCK cells from American Tissue Culture Collection (Manassas, VA, USA) were grown in 1× Dulbecco’s Modified Eagle Medium (Thermo Fisher Scientific, Waltham, MA, USA) supplemented with 5% fetal bovine serum.

### Viruses

We constructed four rgA(H3N2)2013 IAV: (i) a wild-type double-glycosylated virus rg+45/+144 containing PB2, PA, NP, M, and NS genes from A/Puerto Rico/8/34(H1N1), PB1 gene from A/Texas/1/1977(H3N2), and the HA (with 12 NLG sites, including N45 [NSS] and N144 [NSS]) and the NA from A/Switzerland/9715293/2013(H3N2) IAV (NIB-88; NIBSC code: 14/314, National Institute for Biological Standards and Control, Potters Bar, Hertfordshire, EN6 3QG), (ii) single-deleted rg−45/+144, in which asparagine (N) was replaced with serine (S) at residue 45 (N45S) of HA to remove NLG site, (iii) single-deleted rg+45/−144 virus, in which N was replaced with lysine (K) at residue 144 (N144K) of HA, and (iv) double-deleted rg−45/−144 virus containing both substitutions on HA. The amino acid substitutions were chosen to reflect the dominant use by natural isolates. Genes of interest were synthesized by Twist Bioscience (San Francisco, CA, USA) and cloned into the transcription plasmid pCIPolISapIT. Plasmids containing the eight gene segments were transfected into 293T cells at 1 µg each using Lipofectamine 2000 (Invitrogen, Waltham, MA) to produce rg virus. The rescued viruses were grown in 10-day-old embryonated chicken eggs for stocks, which were fully sequenced to ensure the absence of undesired mutations. The infectivity of stock viruses was determined by 50% tissue culture infectious dose (TCID_50_) assays in MDCK cells as described elsewhere (52). Numbering of glycosylation sites is based on the sequence of the mature A/Switzerland/9715293/2013 (H3N2) IAV HA molecule (GISAID [https://gisaid.org], accession number EPI_ISL_207641) without the 16-amino-acid signal sequence. The substitutions introduced at residues 45 and 144 of HA are based on the predominant natural patterns observed with A(H3N2) IAV (Table S1).

### Analysis of viral proteins

Egg-grown rgA(H3N2)2013 IAVs were concentrated and purified through a gradient of 30% to 50% sucrose in PBS, as described previously (53). Prior to glycosylation, biologic and immunologic analyses, viruses were inactivated with β-propiolactone (BPL; Sigma-Aldrich Corp., St. Louis, MO, USA), as described (54). Total protein concentration in the sample was determined using the Bio-Rad protein assay (Bio-Rad, Hercules, CA, USA). Inactivated virus was diluted in radioimmunoprecipitation buffer (Thermo Fisher Scientific) before being washed with acetone for at least 60 min. Then, samples were resuspended in 1× Sample Buffer and heated at 80°C for 10 min. To determine the HA content for binding and immunological assays, we removed N-glycosylated moieties from virus’ glycoprotein by treatment with 500 units of peptide N-glycosidase F (New England Biolabs, Ipswich, MA) for 2 h at 37°C. SDS-PAGE was performed on a 4%–12% NuPAGE Novex Bis–Tris gel (Thermo Fisher Scientific) under non-reducing conditions. Then, the gels were stained with GelCode Blue Safe protein stain (Thermo Fisher Scientific). The imaging was accomplished using UVP ChemStudio SA^2^ BioImaging System (AnalytikJena GmbH, Jena, Germany), and the densities of the HA bands were analyzed with VisionWorks software (AnalytikJena GmbH).

Samples designated for further MS-based analysis underwent subsequent in-gel digestion by reduction and alkylation. Proteins in the bands of interest were selected for digestion with a single or combination of two enzymes, including trypsin, chymotrypsin, Asp-N, Lys-C, and α-lytic protease. Digestions with a single protease were conducted at 37°C overnight. For sequential digestions, the first digestion was performed at 37°C for 5 h, and the second one was conducted at 37°C overnight. Extracted peptides were subjected to MS analysis.

### Glycopeptide analysis

The glycan composition and abundance at each NLG site of HA of proteins were analyzed by nanoflow liquid chromatography coupled with electrospray ionization tandem-mass spectrometry analysis using an Orbitrap Eclipse Tribrid mass spectrometer coupled to an UltiMate3000 RSLC nano chromatography system (Thermo Scientific), as described previously (55). Briefly, the protein digests were injected into C18 column (75 µm i.d. × 15 cm length, 3-µm 100Å particles) and separated using a reversed-phase gradient.

MS data acquisition was accomplished using a signature ion triggered electron-transfer/higher-energy collision dissociation (EThcD) method. The full MS1 precursor scans were acquired by the Orbitrap at a resolution of 120,000, and a data-dependent MS2 scan was acquired using high-energy collision dissociation (HCD) at a resolution of 30,000 and a normalized collision energy of 28%. Three signature ions representing glycan oxonium fragments (HexNAc, m/z 204.0867; HexNAc, 138.0545; or HexNAcHex, 366.1396) were used to trigger the electron-transfer dissociation fragmentation.

MS/MS data were processed using PMi-Byonic (Version 3.4) node within Proteome Discover 2.4 software (Thermo Fisher Scientific). Instrument parameters included the fragmentation of both HCD and EThcD, and the precursor and fragment mass tolerance of 6 and 20 ppm, respectively. Data were searched using an N-glycan 309 mammalian database provided from Byos software (PMi), with the addition of eight more glycans described in a previous report (23). The parameters for enzyme digestion were set to semi-specific with three maximum missed cleavages. Common variable modifications included oxidation of methionine and deamidation of asparagine and glutamine. Carbamidomethylation of cysteine was set as a fixed modification. The abundances of glycopeptide or unoccupied peptide were represented by the ratios of their MS peak areas.

### Binding and hemolysis assays with red blood cells (RBCs)

To determine the receptor binding of concentrated purified rgA(H3N2)2013 viruses, we determined the HA content in each virus sample in SDS-PAGE and then diluted virus in PBS to contain approximately 0.03 μg of the virion HA in the tested volume of 50 μL. HAU titers were determined after incubation of virus with 0.5% turkey RBC for 1 h at 4°C as described elsewhere (52).

To compare thermal stability of the rgA(H3N2)2013 IAVs, we diluted viruses in PBS to 64 HAU/50 µL, incubated them at temperatures ranging from 4°C to 70°C for 20 min, and then, determined HAU titers with 0.5% turkey RBC.

To determine the fusion potential of the rgA(H3N3)2013 IAVs, we examined the hemolytic activities of their HA, as described (33, 34). Briefly, viruses were diluted in PBS to contain 2,000 HAU/100 µL. Fresh turkey RBCs were washed three times with PBS pH 7.2, resuspended in PBS to make 2.0% (v/v) suspension, and pre-warmed for 30 min at 37°C prior to the experiment. Then, 200 µL of these RBCs was mixed with an equal amount of virus in a 96-deep well plate. The mixture was incubated for 30 min at 37°C to allow binding of the virus to RBCs. For triggering HA acidification and hemolysis, 100 µL of sodium acetate buffer (0.5 M, pH ranging from 4.6 to 5.2) was mixed with a suspension of virus and RBCs, and the plate was placed into a 37°C incubator for 30 min. After incubation, the plate was centrifuged at 1,200 rpm for 6 min to separate non-lysed RBCs from supernatant. The supernatant (300 µL) was transferred into a flat-bottom 96-well plate, and released hemoglobin was measured at OD405 nm using the BioTek Epoch 2 microplate spectrophotometer (Agilent Technologies, Santa Clara, CA, USA).

### Viral growth kinetics

In growth kinetics assays, monolayers of MDCK cells in 24-well plates were washed with PBS and infected with tested viruses at MOI 0.001 or 1.0 for 1 h at room temperature (RT). After virus adsorption, cells were washed three times with PBS pH 7.2 to remove nonadherent virus and incubated in infection media supplemented with bovine serum albumin (BSA) and 1 µg/mL acetylated trypsin at 37°C for the duration of the experiment. Tissue culture supernatants were collected at time points ranging from 4 to 72 post-infection (p.i.) hours. The amount of virus in the tissue culture supernatants from infected MDCK cells was determined by TCID_50_ assays in MDCK cells.

### Immune studies in mice

Immunization studies were performed to compare mouse antibody responses generated toward the rgA(H3N2)2013 IAVs. Ten 8-week-old female BALB/c mice (Jackson Laboratories, Bar Harbor, ME, USA) per group were immunized intramuscularly twice at 21-day intervals with 10.0 μg of HA from egg-grown concentrated purified BPL-inactivated virus in sterile PBS, or with sterile PBS as a control. Blood samples were collected on days 0 (before immunization), 35, 43, and 49. Mice were anesthetized with 2.5% inhaled isoflurane (Baxter Healthcare Corporation, Deerfield, IL, USA) prior to all interventions.

### Mouse serum preparation

Sera were treated with receptor-destroying enzyme (RDE; Denka Seiken Co. Ltd., Tokyo, Japan) for 18 h at 37°C (resulting in an initial serum dilution of 1:10), and the enzyme was then heat-inactivated at 55°C for 30 min. RDE-treated sera were adsorbed with packed RBCs at 4°C for 30 min to remove nonspecific agglutinins and then stored at −80°C. Sera were thawed immediately before serological assays.

### Immune assays

ELISA was completed to measure the binding of selected broadly cross-reactive mAbs targeting the stem region (mAb FI6, C66, and C9) and head (mAb C6) of the HA molecule and to measure total antibody titers in the mouse sera. Selected mAbs (C66, C9, and C6) were isolated and produced, and FI6 was produced by Drs. Jason Wilson and Kui Yang (National Center for Immunization & Respiratory Diseases, Influenza Division, Centers for Disease Control & Prevention).

Immobilon 2HB ELISA plates (Thermo Fisher Scientific) were coated overnight at 4°C with 100 HAU/well of the rgA(H3N2)2013 IAVs. Then, plates were washed three times with PBS containing 0.1% Tween 20 (PBS-T) and blocked for 1 h at RT with PBS-T containing 5.0% fetal BSA (Sigma-Aldrich Corp.). Plates were washed again three times with PBS, and 50 μL of threefold serial dilutions (in PBS-T with 0.1% BSA) of mAb or twofold dilutions of 1:1,000 mouse serum samples were added to each well. After 1 h of incubation, plates were washed three times with PBS-T, and then 50 μL of secondary peroxidase-conjugated anti-mouse antibody (Jackson ImmunoResearch, West Grove, PA, USA) diluted (1:5,000) in PBS containing 3% BSA was added to each well. After 30-min incubation, plates were washed five times with PBS-T and developed with TMB (Sigma-Aldrich Corp.) for 10 min at RT. Stop solution (5% sulfuric acid; H_2_SO_4_) was added to wells, and plates were read either at 450 or 490 nm on an Epoch 2 microplate reader (Agilent - BioTek, Santa Clara, CA, USA). GMT antibody titers are given as the reciprocal of the highest dilution which gave an OD_450-490_ value >2 times the average of the background wells.

In HI assays, sera from immunized mice were serially twofold diluted and incubated with four HA of tested virus for 30 min at RT. Agglutination was detected with 0.5% turkey RBCs. HI titers were defined as the reciprocal of the last dilution of serum that completely inhibited hemagglutination.

In neutralization assays, sera from immunized mice were serially twofold diluted and incubated with 100 TCID_50_/100 µL of tested virus for 1 h at 37°C. Then, MDCK cells in 96-well plates were inoculated with the virus antisera mixture and incubated for 48 h at 37°C. The presence of virus was determined by hemagglutination assays with 0.5% turkey RBCs. The minimum detectable limit of either assay was a titer of 10. Samples with titers <10 were assigned a value of 9 for calculating geometric mean titers (GMTs).

### Statistical analyses

Binding of the wild type and glycosylation mutant viruses was compared using unpaired Student’s *t*-tests. Ordinary one-way analysis of variance (ANOVA), followed by Sidak’s multiple comparison test, was used to estimate and compare viruses’ fusion activities, thermal stability, HAU titers, and immunogenicity. A *P*-value of <0.05 was considered significant for all comparisons. Statistical analyses were performed using GraphPad Prism 10.4.2, Excel 2025, and SAS 9.4.

## Data Availability

The HA sequences of A(H3N2) IAV presented in Fig. 1b and c and Table S1 were retrieved from GISAID (GISAID - gisaid.org). Raw LC-MS/MS glycomics data shown in Fig. 2 and Table S2 are deposited on the Mass Spectrometry Interactive Virtual Environment (MassIVE) repository server (https://massive.ucsd.edu/) (accession ID is MassIVE MSV000101765). Other data from this study are included in the article and its supplemental material.

## SUPPLEMENTAL MATERIAL

Fig. S1 jvi.00662-26-s0001.tiff Distances between glycosylation sites of HA.

Fig. S2 jvi.00662-26-s0002.tiff Interaction of A(H3N2)2013 IAV with broadly neutralizing antibodies.

Fig. S3 jvi.00662-26-s0003.tiff Interaction of H3 HA with antibodies.

Table S1 jvi.00662-26-s0004.xlsx Hemagglutinin protein sequences from human A(H3N2) isolates.

Table S2 jvi.00662-26-s0005.docx N-glycosylation profiles of A(H3N2)2013 IAV H3 hemagglutinin derived from wild-type and three glycosylation mutated proteins.

ASM does not own the copyrights to Supplemental Material that may be linked to, or accessed through, an article. The authors have granted ASM a non-exclusive, world-wide license to publish the Supplemental Material files. Please contact the corresponding author directly for reuse.

## References

[1] Waalen K, Kilander A, Dudman SG, Ramos-Ocao R, Hungnes O. 2012. Age-dependent prevalence of antibodies cross-reactive to the influenza A(H3N2) variant virus in sera collected in Norway in 2011. Euro Surveill 17:20170. doi:10.2807/ese.17.19.20170-en 22607964

[2] Hoa LNM, Bryant JE, Choisy M, Nguyet LA, Bao NT, Trang NH, Chuc NTK, Toan TK, Saito T, Takemae N, Horby P, Wertheim H, Fox A. 2015. Population susceptibility to a variant swine-origin influenza virus A(H3N2) in Vietnam, 2011–2012. Epidemiol Infect 143:2959–2964. doi:10.1017/S0950268815000187 PMC4595856 25761949

[3] Park AW, Daly JM, Lewis NS, Smith DJ, Wood JLN, Grenfell BT. 2009. Quantifying the impact of immune escape on transmission dynamics of influenza. Science 326:726–728. doi:10.1126/science.1175980 PMC3800096 19900931

[4] Allen JD, Ross TM. 2018. H3N2 influenza viruses in humans: viral mechanisms, evolution, and evaluation. Hum Vaccin Immunother 14:1840–1847. doi:10.1080/21645515.2018.1462639 PMC6149781 29641358

[5] Broecker F, Liu STH, Sun W, Krammer F, Simon V, Palese P. 2018. Immunodominance of antigenic site B in the hemagglutinin of the current H3N2 influenza virus in humans and mice. J Virol 92:e01100-18. doi:10.1128/JVI.01100-18 PMC6158429 30045991

[6] Bonomo ME, Kim RY, Deem MW. 2019. Modular epitope binding predicts influenza quasispecies dominance and vaccine effectiveness: application to 2018/19 season. Vaccine 37:3154–3158. doi:10.1016/j.vaccine.2019.03.068 31060950

[7] Kim JI, Park MS. 2012. N-linked glycosylation in the hemagglutinin of influenza A viruses. Yonsei Med J 53:886–893. doi:10.3349/ymj.2012.53.5.886 PMC3423856 22869469

[8] York IA, Stevens J, Alymova IV. 2019. Influenza virus N-linked glycosylation and innate immunity. Biosci Rep 39:BSR20171505. doi:10.1042/BSR20171505 PMC6328934 30552137

[9] Ohuchi M, Ohuchi R, Feldmann A, Klenk HD. 1997. Regulation of receptor binding affinity of influenza virus hemagglutinin by its carbohydrate moiety. J Virol 71:8377–8384. doi:10.1128/JVI.71.11.8377-8384.1997 PMC192299 9343193

[10] Marinina VP, Gambarian AS, Bovin NV, Tuzikov AB, Shilov AA, Sinitsyn BV, Matrosovich MN. 2003. The effect of losing glycosylation sites near the receptor-binding region on the receptor phenotype of the human influenza virus H1N1. Mol Biol (Mosk) 37:550–555. doi: 10.1023/A:1024207931650 12815964

[11] Kasson PM, Pande VS. 2008. Structural basis for influence of viral glycans on ligand binding by influenza hemagglutinin. Biophys J 95:L48–L50. doi:10.1529/biophysj.108.141507 PMC2547437 18641068

[12] Yin Y, Zhang X, Qiao Y, Wang X, Su Y, Chen S, Qin T, Peng D, Liu X. 2017. Glycosylation at 11Asn on hemagglutinin of H5N1 influenza virus contributes to its biological characteristics. Vet Res 48:81. doi:10.1186/s13567-017-0484-8 PMC5698926 29162128

[13] Pentiah K, Lees WD, Moss DS, Shepherd AJ. 2015. *N*-linked glycans on influenza A H3N2 hemagglutinin constrain binding of host antibodies, but shielding is limited. Glycobiology 25:124–132. doi:10.1093/glycob/cwu097 25227423

[14] Zost SJ, Parkhouse K, Gumina ME, Kim K, Diaz Perez S, Wilson PC, Treanor JJ, Sant AJ, Cobey S, Hensley SE. 2017. Contemporary H3N2 influenza viruses have a glycosylation site that alters binding of antibodies elicited by egg-adapted vaccine strains. Proc Natl Acad Sci USA 114:12578–12583. doi:10.1073/pnas.1712377114 PMC5703309 29109276

[15] Qiu Y, Stegalkina S, Zhang J, Boudanova E, Park A, Zhou Y, Prabakaran P, Pougatcheva S, Ustyugova IV, Vogel TU, Mundle ST, Oomen R, Delagrave S, Ross TM, Kleanthous H, Qiu H. 2020. Mapping of a novel H3-specific broadly neutralizing monoclonal antibody targeting the hemagglutinin globular head isolated from an elite influenza virus-immunized donor exhibiting serological breadth. J Virol 94:e01035-19. doi:10.1128/JVI.01035-19 PMC7158729 31826999

[16] Ekiert DC, Kashyap AK, Steel J, Rubrum A, Bhabha G, Khayat R, Lee JH, Dillon MA, O’Neil RE, Faynboym AM, Horowitz M, Horowitz L, Ward AB, Palese P, Webby R, Lerner RA, Bhatt RR, Wilson IA. 2012. Cross-neutralization of influenza A viruses mediated by a single antibody loop. Nature 489:526–532. doi:10.1038/nature11414 PMC3538848 22982990

[17] Kornfeld R, Kornfeld S. 1985. Assembly of asparagine-linked oligosaccharides. Annu Rev Biochem 54:631–664. doi:10.1146/annurev.bi.54.070185.003215 3896128

[18] Schwarz F, Aebi M. 2011. Mechanisms and principles of N-linked protein glycosylation. Curr Opin Struct Biol 21:576–582. doi:10.1016/j.sbi.2011.08.005 21978957

[19] Gavel Y, von Heijne G. 1990. Sequence differences between glycosylated and non-glycosylated Asn-X-Thr/Ser acceptor sites: implications for protein engineering. Protein Eng Des Sel 3:433–442. doi:10.1093/protein/3.5.433 PMC7529082 2349213

[20] Ruiz-Canada C, Kelleher DJ, Gilmore R. 2009. Cotranslational and posttranslational N-glycosylation of polypeptides by distinct mammalian OST isoforms. Cell 136:272–283. doi:10.1016/j.cell.2008.11.047 PMC2859625 19167329

[21] Chang D, Hackett WE, Zhong L, Wan XF, Zaia J. 2020. Measuring site-specific glycosylation similarity between influenza A virus variants with statistical certainty. Mol Cell Proteomics 19:1533–1545. doi:10.1074/mcp.RA120.002031 PMC8143645 32601173

[22] Li J, Liu S, Gao Y, Tian S, Yang Y, Ma N. 2021. Comparison of N-linked glycosylation on hemagglutinins derived from chicken embryos and MDCK cells: a case of the production of a trivalent seasonal influenza vaccine. Appl Microbiol Biotechnol 105:3559–3572. doi:10.1007/s00253-021-11247-5 PMC8088833 33937925

[23] Alymova IV, Cipollo JF, Parsons LM, Music N, Kamal RP, Tzeng W-P, Goldsmith CS, Contessa JN, Hartshorn KL, Wilson JR, Zeng H, Gansebom S, York IA. 2022. Aberrant cellular glycosylation may increase the ability of influenza viruses to escape host immune responses through modification of the viral glycome. mBio 13:e0298321. doi:10.1128/mbio.02983-21 PMC9040841 35285699

[24] Weis W, Brown JH, Cusack S, Paulson JC, Skehel JJ, Wiley DC. 1988. Structure of the influenza virus haemagglutinin complexed with its receptor, sialic acid. Nature 333:426–431. doi:10.1038/333426a0 3374584

[25] Bullough PA, Hughson FM, Skehel JJ, Wiley DC. 1994. Structure of influenza haemagglutinin at the pH of membrane fusion. Nature 371:37–43. doi:10.1038/371037a0 8072525

[26] El Moussi A, Ben Hadj Kacem MA, Slim A. 2014. Loss and gain of N-linked glycosylation sites in globular head and stem of HA found in A/H3N2 flu fatal and severe cases during 2013 Tunisia flu seasonal survey. Virus Genes 48:189–192. doi:10.1007/s11262-013-0993-0 24174280

[27] Bragstad K, Nielsen LP, Fomsgaard A. 2008. The evolution of human influenza A viruses from 1999 to 2006: a complete genome study. Virol J 5:40. doi:10.1186/1743-422X-5-40 PMC2311284 18325125

[28] Ushirogawa H, Naito T, Tokunaga H, Tanaka T, Nakano T, Terada K, Ohuchi M, Saito M. 2016. Re-emergence of H3N2 strains carrying potential neutralizing mutations at the N-linked glycosylation site at the hemagglutinin head, post the 2009 H1N1 pandemic. BMC Infect Dis 16:380. doi:10.1186/s12879-016-1738-1 PMC4977674 27503338

[29] Rogers GN, D’Souza BL. 1989. Receptor binding properties of human and animal H1 influenza virus isolates. Virology 173:317–322. doi:10.1016/0042-6822(89)90249-3 2815586

[30] Killian ML. 2020. Hemagglutination assay for influenza virus. Methods Mol Biol 2123:3–10. doi:10.1007/978-1-0716-0346-8_1 32170676

[31] Li R, Gao J, Wang L, Gui M, Xiang Y. 2024. Multivalent interactions between fully glycosylated influenza virus hemagglutinins mediated by glycans at distinct N-glycosylation sites. Npj Viruses 2:48. doi:10.1038/s44298-024-00059-9 PMC11721446 40295773

[32] Russell CJ. 2014. Acid-induced membrane fusion by the hemagglutinin protein and its role in influenza virus biology. Curr Top Microbiol Immunol 385:93–116. doi:10.1007/82_2014_393 PMC7122338 25007844

[33] Doms RW, Gething MJ, Henneberry J, White J, Helenius A. 1986. Variant influenza virus hemagglutinin that induces fusion at elevated pH. J Virol 57:603–613. doi:10.1128/JVI.57.2.603-613.1986 PMC252775 3003392

[34] Krenn BM, Egorov A, Romanovskaya-Romanko E, Wolschek M, Nakowitsch S, Ruthsatz T, Kiefmann B, Morokutti A, Humer J, Geiler J, Cinatl J, Michaelis M, Wressnigg N, Sturlan S, Ferko B, Batishchev OV, Indenbom AV, Zhu R, Kastner M, Hinterdorfer P, Kiselev O, Muster T, Romanova J. 2011. Single HA2 mutation increases the infectivity and immunogenicity of a live attenuated H5N1 intranasal influenza vaccine candidate lacking NS1. PLoS One 6:e18577. doi:10.1371/journal.pone.0018577 PMC3072404 21490925

[35] Li Y, Liu D, Wang Y, Su W, Liu G, Dong W. 2021. The importance of glycans of viral and host proteins in enveloped virus infection. Front Immunol 12:638573. doi:10.3389/fimmu.2021.638573 PMC8116741 33995356

[36] Corti D, Voss J, Gamblin SJ, Codoni G, Macagno A, Jarrossay D, Vachieri SG, Pinna D, Minola A, Vanzetta F, Silacci C, Fernandez-Rodriguez BM, Agatic G, Bianchi S, Giacchetto-Sasselli I, Calder L, Sallusto F, Collins P, Haire LF, Temperton N, Langedijk JPM, Skehel JJ, Lanzavecchia A. 2011. A neutralizing antibody selected from plasma cells that binds to group 1 and group 2 influenza A hemagglutinins. Science 333:850–856. doi:10.1126/science.1205669 21798894

[37] Altman MO, Angel M, Košík I, Trovão NS, Zost SJ, Gibbs JS, Casalino L, Amaro RE, Hensley SE, Nelson MI, Yewdell JW. 2019. Human influenza A virus hemagglutinin glycan evolution follows a temporal pattern to a glycan limit. mBio 10:e00204-19. doi:10.1128/mBio.00204-19 PMC6445938 30940704

[38] Malaby HLH, Kobertz WR. 2014. The middle X residue influences cotranslational N-glycosylation consensus site skipping. Biochemistry 53:4884–4893. doi:10.1021/bi500681p PMC4372077 25029371

[39] Nita-Lazar M, Wacker M, Schegg B, Amber S, Aebi M. 2005. The N-X-S/T consensus sequence is required but not sufficient for bacterial N-linked protein glycosylation. Glycobiology 15:361–367. doi:10.1093/glycob/cwi019 15574802

[40] Kiso M, Ozawa M, Le MTQ, Imai H, Takahashi K, Kakugawa S, Noda T, Horimoto T, Kawaoka Y. 2011. Effect of an asparagine-to-serine mutation at position 294 in neuraminidase on the pathogenicity of highly pathogenic H5N1 influenza A virus. J Virol 85:4667–4672. doi:10.1128/JVI.00047-11 PMC3126218 21367898

[41] Russier M, Yang G, Rehg JE, Wong S-S, Mostafa HH, Fabrizio TP, Barman S, Krauss S, Webster RG, Webby RJ, Russell CJ. 2016. Molecular requirements for a pandemic influenza virus: An acid-stable hemagglutinin protein. Proc Natl Acad Sci USA 113:1636–1641. doi:10.1073/pnas.1524384113 PMC4760800 26811446

[42] Koerner I, Matrosovich MN, Haller O, Staeheli P, Kochs G. 2012. Altered receptor specificity and fusion activity of the haemagglutinin contribute to high virulence of a mouse-adapted influenza A virus. J Gen Virol 93:970–979. doi:10.1099/vir.0.035782-0 22258863

[43] Mair CM, Ludwig K, Herrmann A, Sieben C. 2014. Receptor binding and pH stability — How influenza A virus hemagglutinin affects host-specific virus infection. Biochim Biophys Acta 1838:1153–1168. doi:10.1016/j.bbamem.2013.10.004 24161712

[44] Hasegawa K, Hu C, Nakamura T, Marks JD, Russell SJ, Peng K-W. 2007. Affinity thresholds for membrane fusion triggering by viral glycoproteins. J Virol 81:13149–13157. doi:10.1128/JVI.01415-07 PMC2169077 17804513

[45] Negi G, Sharma A, Dey M, Dhanawat G, Parveen N. 2022. Membrane attachment and fusion of HIV-1, influenza A, and SARS-CoV-2: resolving the mechanisms with biophysical methods. Biophys Rev 14:1109–1140. doi:10.1007/s12551-022-00999-7 PMC9552142 36249860

[46] Mair CM, Meyer T, Schneider K, Huang Q, Veit M, Herrmann A. 2014. A histidine residue of the influenza virus hemagglutinin controls the pH dependence of the conformational change mediating membrane fusion. J Virol 88:13189–13200. doi:10.1128/JVI.01704-14 PMC4249083 25187542

[47] Alymova IV, York IA, Air GM, Cipollo JF, Gulati S, Baranovich T, Kumar A, Zeng H, Gansebom S, McCullers JA. 2016. Glycosylation changes in the globular head of H3N2 influenza hemagglutinin modulate receptor binding without affecting virus virulence. Sci Rep 6:36216. doi:10.1038/srep36216 PMC5086918 27796371

[48] Wanzeck K, Boyd KL, McCullers JA. 2011. Glycan shielding of the influenza virus hemagglutinin contributes to immunopathology in mice. Am J Respir Crit Care Med 183:767–773. doi:10.1164/rccm.201007-1184OC PMC3159075 20935106

[49] de Vries RP, Smit CH, de Bruin E, Rigter A, de Vries E, Cornelissen L, Eggink D, Chung NPY, Moore JP, Sanders RW, Hokke CH, Koopmans M, Rottier PJM, de Haan CAM. 2012. Glycan-dependent immunogenicity of recombinant soluble trimeric hemagglutinin. J Virol 86:11735–11744. doi:10.1128/JVI.01084-12 PMC3486279 22915811

[50] Huang QJ, Kim R, Song K, Grigorieff N, Munro JB, Schiffer CA, Somasundaran M. 2025. Virion-associated influenza hemagglutinin clusters upon sialic acid binding visualized by cryoelectron tomography. Proc Natl Acad Sci USA 122:e2426427122. doi:10.1073/pnas.2426427122 PMC12037027 40244672

[51] Thomès L, Joeres R, Akdeniz Z, Bojar D. 2025. GlyContact analyzes glycan 3D structures at scale. Nat Commun 16:11136. doi:10.1038/s41467-025-67590-y PMC12706034 41387443

[52] Condit RC. 2007. Principles of virology, p 25–57. Lippincott Williams & Wilkins.

[53] Thompson SD, Laver WG, Murti KG, Portner A. 1988. Isolation of a biologically active soluble form of the hemagglutinin-neuraminidase protein of Sendai virus. J Virol 62:4653–4660. doi:10.1128/JVI.62.12.4653-4660.1988 PMC253578 2846877

[54] Sasaki Y, Yoshino N, Sato S, Muraki Y. 2016. Analysis of the beta-propiolactone sensitivity and optimization of inactivation methods for human influenza H3N2 virus. J Virol Methods 235:105–111. doi:10.1016/j.jviromet.2016.04.013 27142111

[55] Wang D, Zhang Z, Baudys J, Haynes C, Osman SH, Zhou B, Barr JR, Gumbart JC. 2024. Enhanced surface accessibility of SARS-CoV-2 Omicron spike protein due to an altered glycosylation profile. ACS Infect Dis 10:2032–2046. doi:10.1021/acsinfecdis.4c00015 PMC11184558 38728322

