# Supplementary material for: N-linked glycosylation sites with low occupancy support sustained circulation of the A(H3N2) influenza A virus in the human population
Source: J Virol. 2026 Aug 28;100(9):e00662-26. doi: 10.1128/jvi.00662-26 (PMC13595915; doi:10.1128/jvi.00662-26)
Supplement: Supplementary file 1 [file jvi.00662-26-s0005.docx]

| **Table S2.** N-glycosylation profiles of A(H3N2)2013 IAV H3 hemagglutinin derived from wild-type and three glycosylation mutated proteins.^a^ | | | | | |
| --- | --- | --- | --- | --- | --- |
| **Glycosylation site** | **Glycan composition** | **Relative glycan abundance (% ± SD)** | | | |
|  |  | wild type | N45S  mutant | N144K  mutant | N45S/N144K mutant |
| 8 (NSTA) | *HexNAc(2)Hex(3)* | 1.9 ± 0.4 | 2.5 ± 0.4 | 2.1 ± 0.3 | 2.8 ± 0.5 |
|  | *HexNAc(2)Hex(4)* | 5.3 ± 0.9 | 5.1 ± 1.6 | 3.0 ± 0.1 | 6.3 ± 1.3 |
|  | *HexNAc(2)Hex(5)* | 37.3 ± 5.3 | 35.2 ± 1.9 | 40.0 ± 5.3 | 31.7 ± 3.7 |
|  | *HexNAc(2)Hex(6)* | 12.3 ± 2.1 | 14.1 ± 0.3 | 13.6 ± 2.9 | 17.9 ± 2.9 |
|  | *HexNAc(2)Hex(7)* | 4.0 ± 0.8 | 4.6 ± 0.3 | 5.6 ± 1.0 | 6.5 ± 1.0 |
|  | *HexNAc(3)Hex(5)* | 1.8 ± 0.3 | 1.4 ± 0.1 | 1.3 ± 0.2 | 1.0 ± 0.3 |
|  | *HexNAc(3)Hex(6)* | 14.1 ± 1.3 | 13.2 ± 0.7 | 11.9 ± 1.6 | 12.6 ± 0.8 |
|  | *HexNAc(3)Hex(6)Fuc(1)* | 1.7 ± 0.1 | 0.8 ± 0.2 | 1.6 ± 0.3 | 1.8 ± 0.3 |
|  | *HexNAc(4)Hex(5)* | 2.4 ± 0.2 | 3.1 ± 0.4 | 2.0 ± 0.5 | 1.5 ± 0.3 |
|  | *HexNAc(4)Hex(5)Fuc(1)* | 10.1 ± 1.7 | 9.8 ± 0.4 | 5.9 ± 1.3 | 5.0 ± 1.2 |
|  | *HexNAc(4)Hex(5)Fuc(2)* | 1.5 ± 0.2 | 0.6 ± 0.1 | 0.6 ± 0.2 | 0.8 ± 0.2 |
|  | *HexNAc(5)Hex(5)* | 0.0 ± 0.0 | 0.9 ± 0.2 | 0.8 ± 0.1 | 0.8 ± 0.1 |
|  | *HexNAc(5)Hex(5)Fuc(1)* | 2.3 ± 0.8 | 2.2 ± 0.6 | 3.5 ± 0.8 | 4.0 ± 0.3 |
| Site occupancy |  | 94.6 ± 1.1 | 93.4 ± 0.6 | 91.8 ± 1.1 | 92.6 ± 1.0 |
|  |  |  |  |  |  |
| 22 (NGTI) | *HexNAc(4)Hex(4)Fuc(1)NeuAc(1)* | 6.0 ± 2.3 | 3.3 ± 0.4 | 4.0 ± 0.1 | 2.4 ± 0.6 |
|  | *HexNAc(4)Hex(4)Fuc(2)NeuAc(1)* | ND | ND | ND | 7.5 ± 0.4 |
|  | *HexNAc(4)Hex(5)Fuc(1)* | ND | 8.4 ± 0.7 | 12.0 ± 1.2 | 7.4 ± 0.1 |
|  | *HexNAc(4)Hex(5)Fuc(2)NeuAc(1)* | 1.4 ± 0.3 | 1.1 ± 0.3 | 1.1 ± 0.2 | ND |
|  | *HexNAc(4)Hex(5)Fuc(3)* | ND | 1.1 ± 0.3 | 3.3 ± 0.9 | ND |
|  | *HexNAc(4)Hex(5)Fuc(3)NeuAc(1)* | 0.5 ± 0.0 | ND | ND | ND |
|  | *HexNAc(5)Hex(4)Fuc(1)NeuAc(1)* | 5.2 ± 1.7 | ND | ND | 4.1 ± 0.6 |
|  | *HexNAc(5)Hex(4)Fuc(2)NeuAc(1)* | ND | ND | ND | 2.1 ± 0.3 |
|  | *HexNAc(5)Hex(4)Fuc(2)NeuGc(1)* | 2.6 ± 0.1 | 1.4 ± 0.4 | ND | 1.9 ± 0.1 |
|  | *HexNAc(5)Hex(4)NeuAc(1)* | 12.3 ± 2.2 | 9.8 ± 0.9 | ND | 10.7 ± 3.7 |
|  | *HexNAc(5)Hex(5)* | 0.0 ± 0.0 | 0.0 ± 0.0 | 4.7 ± 1.1 | ND |
|  | *HexNAc(5)Hex(5)Fuc(1)* | 33.7 ± 8.6 | 28.0 ± 3.1 | 34.1 ± 8.2 | 24.7 ± 1.4 |
|  | *HexNAc(5)Hex(5)Fuc(2)* | 0.0 ± 0.0 | 9.8 ± 1.4 | 0.0 ± 0.0 | 11.2 ± 0.4 |
|  | *HexNAc(5)Hex(5)Fuc(3)* | 5.8 ± 0.4 | 1.7 ± 0.6 | 0.0 ± 0.0 | 0.0 ± 0.0 |
|  | *HexNAc(5)Hex(5)NeuAc(1)* | 5.4 ± 0.1 | 6.3 ± 0.2 | 6.9 ± 1.1 | 3.9 ± 1.3 |
|  | *HexNAc(5)Hex(6)* | ND | ND | 0.8 ± 0.1 | ND |
|  | *HexNAc(5)Hex(6)Fuc(1)* | 6.2 ± 1.3 | 8.3 ± 0.5 | 6.7 ± 1.0 | 4.6 ± 0.3 |
|  | *HexNAc(5)Hex(6)Fuc(2)* | 2.4 ± 0.5 | ND | ND | 1.6 ± 0.2 |
|  | *HexNAc(5)Hex(6)Fuc(2)NeuAc(1)* | 0.2 ± 0.0 | 0.5 ± 0.1 | ND | ND |
|  | *HexNAc(6)Hex(5)Fuc(1)NeuAc(1)* | 0.6 ± 0.1 | ND | ND | 0.7 ± 0.2 |
|  | *HexNAc(6)Hex(6)Fuc(1)* | 3.3 ± 0.2 | 3.1 ± 0.6 | 4.9 ± 0.2 | 3.9 ± 0.7 |
|  | *HexNAc(6)Hex(6)NeuAc(1)* | 2.5 ± 0.2 | 3.4 ± 0.2 | 2.7 ± 0.9 | 2.3 ± 0.1 |
|  | *HexNAc(6)Hex(7)Fuc(1)* | 1.7 ± 0.5 | 2.7 ± 0.1 | 3.1 ± 0.7 | 1.9 ± 0.2 |
|  | *HexNAc(6)Hex(7)Fuc(4)* | ND | 0.3 ± 0.1 | ND | 0.3 ± 0.1 |
|  | *HexNAc(6)Hex(7)NeuAc(1)* | 0.8 ± 0.0 | 1.0 ± 0.0 | 1.0 ± 0.0 | 0.3 ± 0.0 |
|  | *HexNAc(7)Hex(7)Fuc(1)* | 0.7 ± 0.1 | 1.6 ± 0.1 | 1.2 ± 0.4 | 1.3 ± 0.2 |
| Site occupancy |  | 91.1 ± 1.0 | 92.1 ± 0.5 | 86.6 ± 1.0 | 92.8 ± 0.6 |
|  |  |  |  |  |  |
| 38 (NATE) | *HexNAc(2)Hex(4)* | ND | 0.4 ± 0.0 | 0.5 ± 0.1 | 0.4 ± 0.0 |
|  | *HexNAc(2)Hex(5)* | 29.5 ± 3.9 | 21.4 ± 3.3 | 27.9 ± 6.5 | 26.0 ± 4.1 |
|  | *HexNAc(2)Hex(6)* | 18.3 ± 0.7 | 17.8 ± 2.4 | 17.7 ± 0.4 | 19.7 ± 2.1 |
|  | *HexNAc(2)Hex(7)* | 10.6 ± 0.7 | 15.4 ± 1.6 | 12.7 ± 0.3 | 14.8 ± 3.8 |
|  | *HexNAc(2)Hex(8)* | ND | 0.3 ± 0.1 | ND | 0.2 ± 0.0 |
|  | *HexNAc(3)Hex(4)* | ND | 4.1 ± 0.8 | 4.2 ± 0.3 | 2.9 ± 0.6 |
|  | *HexNAc(3)Hex(5)* | 7.1 ± 0.7 | 4.6 ± 0.8 | 4.7 ± 1.4 | 2.9 ± 0.6 |
|  | *HexNAc(3)Hex(5)Fuc(1)* | 4.8 ± 0.2 | ND | ND | ND |
|  | *HexNAc(3)Hex(6)* | 20.4 ± 2.3 | 20.6 ± 2.8 | 17.3 ± 2.0 | 13.5 ± 3.3 |
|  | *HexNAc(3)Hex(6)Fuc(1)* | ND | 6.9 ± 1.1 | 10.6 ± 2.8 | 11.4 ± 3.7 |
|  | *HexNAc(4)Hex(5)* | 6.7 ± 1.3 | 2.7 ± 0.6 | 3.5 ± 1.1 | 2.4 ± 0.7 |
|  | *HexNAc(4)Hex(5)Fuc(1)* | ND | 2.0 ± 0.2 | ND | 0.7 ± 0.1 |
|  | *HexNAc(4)Hex(6)* | 1.3 ± 0.4 | 1.6 ± 0.5 | 0.9 ± 0.2 | 2.5 ± 0.8 |
|  | *HexNAc(4)Hex(7)* | ND | 1.3 ± 0.5 | *ND* | 1.7 ± 0.0 |
|  | *HexNAc(5)Hex(5)* | 1.3 ± 0.2 | ND | ND | ND |
|  | *HexNAc(5)Hex(5)Fuc(1)* | ND | 0.2 ± 0.0 | ND | 0.5 ± 0.1 |
|  | *HexNAc(5)Hex(6)* | ND | 0.8 ± 0.1 | ND | 0.6 ± 0.1 |
| Site occupancy |  | 100.0 ± 1.2 | 100.0 ± 1.0 | 100.0 ± 1.5 | 100.0 ± 1.3 |
|  |  |  |  |  |  |
| 45 (NSSI)^b^ | *HexNAc(2)Hex(5)* | 0.7 ± 0.6 | ND | 0.5 ± 0.3 | ND |
|  | *HexNAc(2)Hex(6)* | 1.1 ± 0.9 | ND | 0.7 ± 0.5 | ND |
|  | *HexNAc(2)Hex(7)* | 1.7 ± 1.4 | ND | 1.2 ± 0.0 | ND |
|  | *HexNAc(3)Hex(6)* | 0.4 ± 0.2 | ND | 0.4 ± 0.1 | ND |
| Site occupancy |  | 3.9 ± 0.8 | 0.0 ± 0.0 | 2.8 ± 0.2 | 0.0 ± 0.0 |
|  |  |  |  |  |  |
| 63 (NCTL) | *HexNAc(4)Hex(4)* | ND | ND | 3.0 ± 1.0 | ND |
|  | *HexNAc(4)Hex(5)Fuc(1)* | ND | 8.5 ± 0.4 | ND | ND |
|  | *HexNAc(5)Hex(5)* | 54.2 ± 8.5 | 57.8 ± 1.9 | 97.0 ± 24.5 | ND |
|  | *HexNAc(5)Hex(5)Fuc(1)* | 45.8 ± 2.6 | 31.0 ± 2.4 | ND | 100.0 ± 10.0 |
|  | *HexNAc(5)Hex(6)* | ND | 2.6 ± 0.7 | ND | ND |
| Site occupancy |  | 100.0 ± 5.6 | 100.0 ± 1.4 | 100.0 ± 12.7 | 100.0 ± 10.0 |
|  |  |  |  |  |  |
| 122 (NESF) | *HexNAc(1)Fuc(1)* | 0.2 ± 0.0 | 0.2 ± 0.0 | ND | ND |
|  | *HexNAc(5)Hex(4)* | ND | 0.3 ± 0.0 | ND | ND |
|  | *HexNAc(5)Hex(5)* | ND | 0.5 ± 0.1 | ND | 0.3 ± 0.1 |
|  | *HexNAc(5)Hex(6)* | ND | 0.3 ± 0.0 | ND | 0.3 ± 0.1 |
| Site occupancy |  | 0.2 ± 0.0 | 1.3 ± 0.1 | 0.0 ± 0.0 | 0.6 ± 0.1 |
|  |  |  |  |  |  |
| 133 (NGTS) | *HexNAc(1)* | 0.2 ± 0.0 | 0.1 ± 0.0 | 0.2 ± 0.0 | ND |
|  | *HexNAc(2)Hex(3)* | ND | ND | 1.0 ± 0.1 | 1.1 ± 0.3 |
|  | *HexNAc(2)Hex(4)* | 0.4 ± 0.0 | 0.5 ± 0.1 | 0.8 ± 0.0 | 1.0 ± 0.1 |
|  | *HexNAc(2)Hex(5)* | 23.2 ± 8.6 | 20.4 ± 2.6 | 17.3 ± 0.5 | 19.4 ± 0.4 |
|  | *HexNAc(2)Hex(6)* | 11.4 ± 2.7 | 16.4 ± 2.2 | 17.5 ± 2.6 | 18.4 ± 3.2 |
|  | *HexNAc(2)Hex(7)* | 33.6 ± 0.6 | 35 ± 5.9 | 57.5 ± 0.7 | 53.5 ± 14.6 |
|  | *HexNAc(2)Hex(8)* | 0.6 ± 0.1 | ND | 0.5 ± 0.1 | ND |
|  | *HexNAc(3)Hex(4)* | 1.9 ± 0.2 | 2.4 ± 0.4 | ND | ND |
|  | *HexNAc(3)Hex(4)Fuc(1)* | 2.1 ± 0.1 | 0.9 ± 0.2 | ND | ND |
|  | *HexNAc(3)Hex(5)* | 0.8 ± 0.1 | 0.7 ± 0.0 | ND | ND |
|  | *HexNAc(3)Hex(6)* | 5.5 ± 1.7 | 6.1 ± 0.8 | 2.6 ± 0.7 | 2.4 ± 0.6 |
|  | *HexNAc(4)Hex(5)* | 3.2 ± 0.9 | 4.4 ± 0.8 | ND | 0.8 ± 0.2 |
|  | *HexNAc(4)Hex(5)Fuc(1)* | 1.2 ± 0.2 | 0.6 ± 0.0 | ND | ND |
|  | *HexNAc(4)Hex(6)* | 2.6 ± 0.5 | 2.3 ± 0.2 | ND | ND |
|  | *HexNAc(4)Hex(7)* | 4.5 ± 1.4 | 3.1 ± 0.1 | ND | 0.3 ± 0.1 |
|  | *HexNAc(5)Hex(5)* | 4.4 ± 1.1 | 3.5 ± 0.5 | ND | ND |
| Site occupancy |  | 95.5 ± 1.2 | 96.4 ± 1.0 | 97.4 ± 0.6 | 96.8 ± 2.4 |
|  |  |  |  |  |  |
| 144 (NSSF) | *HexNAc(4)Hex(5)Fuc(1)* | 1.7 ± 0.5 | 1.3 ± 0.4 | ND | ND |
|  | *HexNAc(5)Hex(5)* | 0.3 ± 0.0 | 0.3 ± 0.0 | ND | ND |
|  | *HexNAc(5)Hex(5)Fuc(1)* | 5.3 ± 1.1 | 3.7 ± 1.3 | ND | ND |
| Site occupancy |  | 7.3 ± 0.8 | 5.3 ± 0.9 | 0.0 ± 0.0 | 0.0 ± 0.0 |
|  |  |  |  |  |  |
| 165 (NVTM) | *HexNAc(2)Hex(5)* | 1.7 ± 0.4 | 2.2 ± 0.5 | 2.6 ± 0.8 | 1.6 ± 0.4 |
|  | *HexNAc(2)Hex(6)* | 0.6 ± 0.0 | 0.5 ± 0.2 | 0.8 ± 0.1 | 1.5 ± 0.2 |
|  | *HexNAc(2)Hex(7)* | 3.3 ± 0.8 | 3.9 ± 0.7 | 3.9 ± 0.9 | 4.5 ± 1.0 |
|  | *HexNAc(2)Hex(8)* | 47.5 ± 8.4 | 49.3 ± 2.9 | 46.6 ± 3.0 | 46.9 ± 1.7 |
|  | *HexNAc(2)Hex(9)* | 36.0 ± 4.9 | 35.6 ± 5.9 | 37.7 ± 3.1 | 39.1 ± 2.5 |
|  | *HexNAc(2)Hex(10)* | 6.9 ± 2.1 | 3.6 ± 0.6 | 5.7 ± 2.1 | 6.4 ± 1.3 |
|  | *HexNAc(3)Hex(6)* | 4.0 ± 1.2 | 4.9 ± 0.7 | 2.8 ± 0.7 | ND |
| Site occupancy |  | 100.0 ± 2.5 | 100.0 ± 1.6 | 100.0 ± 1.5 | 100.0 ± 1.2 |
|  |  |  |  |  |  |
| 246 (NSTG) | *HexNAc(2)Hex(5)* | 1.0 ± 0.3 | 1.3 ± 0.4 | 0.9 ± 0.2 | 1.0 ± 0.3 |
|  | *HexNAc(2)Hex(6)* | 0.5 ± 0.2 | 0.5 ± 0.1 | 0.7 ± 0.1 | 0.7 ± 0.1 |
|  | *HexNAc(2)Hex(7)* | 0.4 ± 0.1 | 0.4 ± 0.1 | 0.4 ± 0.1 | 0.3 ± 0.1 |
|  | *HexNAc(2)Hex(8)* | 4.1 ± 0.6 | 3.7 ± 0.1 | 3.4 ± 0.4 | 3.6 ± 0.2 |
|  | *HexNAc(2)Hex(9)* | 87.8 ± 1.2 | 87.6 ± 0.3 | 86.6 ± 2.7 | 86.6 ± 2.0 |
|  | *HexNAc(2)Hex(10)* | 5.6 ± 0.4 | 5.7 ± 0.3 | 7.2 ± 0.5 | 7.0 ± 1.2 |
|  | *HexNAc(4)Hex(4)Fuc(2)NeuAc(1)* | 0.1 ± 0.0 | 0.1 ± 0.0 | ND | 0.1 ± 0.0 |
|  | *HexNAc(6)Hex(4)* | 0.1 ± 0.0 | 0.3 ± 0.1 | 0.2 ± 0.1 | 0.2 ± 0.0 |
| Site occupancy |  | 99.6 ± 0.4 | 99.5 ± 0.2 | 99.3 ± 0.6 | 99.5 ± 0.5 |
|  |  |  |  |  |  |
| 285 (NGSI) | *HexNAc(2)Hex(4)* | 0.6 ± 0.0 | 0.6 ± 0.1 | 0.3 ± 0.0 | 0.3 ± 0.0 |
|  | *HexNAc(2)Hex(5)* | 1.8 ± 0.3 | 3.1 ± 0.5 | 1.2 ± 0.3 | 2.6 ± 0.4 |
|  | *HexNAc(2)Hex(6)* | 4.5 ± 0.7 | 7.2 ± 1.1 | 3.1 ± 0.7 | 6.4 ± 0.6 |
|  | *HexNAc(2)Hex(7)* | 32.1 ± 4.1 | 44.4 ± 2.3 | 28.9 ± 4.2 | 39.2 ± 4.4 |
|  | *HexNAc(2)Hex(8)* | 9.3 ± 2.6 | 5.0 ± 0.5 | 10.2 ± 2.6 | 3.9 ± 0.1 |
|  | *HexNAc(2)Hex(9)* | 13.1 ± 3.1 | 0.7 ± 0.1 | 12.8 ± 3.1 | 0.4 ± 0.1 |
|  | *HexNAc(6)Hex(5)Fuc(1)* | 0.6 ± 0.2 | 0.8 ± 0.1 | ND | 0.9 ± 0.0 |
|  | *HexNAc(6)Hex(6)Fuc(1)* | 1.6 ± 0.3 | 1.8 ± 0.3 | 1.3 ± 0.0 | 2.5 ± 0.1 |
|  | *HexNAc(6)Hex(7)Fuc(1)* | 12.2 ± 0.7 | 15.3 ± 1.4 | 10.0 ± 0.6 | 17.2 ± 1.1 |
|  | *HexNAc(6)Hex(8)Fuc(1)* | 1.4 ± 0.1 | 0.8 ± 0.1 | 1.8 ± 0.4 | 1.0 ± 0.1 |
| Site occupancy |  | 77.1 ± 1.2 | 79.7 ± 0.7 | 69.6 ± 1.1 | 74.4 ± 0.7 |
|  |  |  |  |  |  |
| 483 (NGTY) | *HexNAc(2)Hex(5)* | 0.2 ± 0.1 | 0.3 ± 0.0 | 0.6 ± 0.2 | 0.7 ± 0.1 |
|  | *HexNAc(3)Hex(4)Fuc(1)* | 0.4 ± 0.1 | 0.7 ± 0.2 | 1.0 ± 0.2 | 1.0 ± 0.2 |
|  | *HexNAc(3)Hex(4)Fuc(2)* | 0.3 ± 0.1 | 0.2 ± 0.0 | 0.3 ± 0.1 | 0.4 ± 0.1 |
|  | *HexNAc(3)Hex(5)* | 0.2 ± 0.0 | 0.3 ± 0.0 | 0.4 ± 0.1 | 0.3 ± 0.1 |
|  | *HexNAc(3)Hex(5)Fuc(1)* | 0.3 ± 0.1 | 0.4 ± 0.1 | 0.6 ± 0.1 | 0.5 ± 0.1 |
|  | *HexNAc(3)Hex(6)* | 0.7 ± 0.1 | 1.4 ± 0.3 | 1.5 ± 0.2 | 1.8 ± 0.3 |
|  | *HexNAc(3)Hex(6)Fuc(1)* | 1.0 ± 0.2 | 0.8 ± 0.1 | 1.5 ± 0.3 | 1.7 ± 0.1 |
|  | *HexNAc(4)Hex(4)Fuc(1)* | 0.1 ± 0.0 | 0.2 ± 0.0 | 0.3 ± 0.0 | 0.2 ± 0.0 |
|  | *HexNAc(4)Hex(4)Fuc(2)NeuAc(1)* | ND | 0.5 ± 0.1 | 0.2 ± 0.0 | 0.3 ± 0.0 |
|  | *HexNAc(4)Hex(5)* | 1.0 ± 0.1 | 1.7 ± 0.2 | 1.6 ± 0.0 | 1.0 ± 0.0 |
|  | *HexNAc(4)Hex(5)Fuc(1)* | 23.5 ± 1.4 | 30.7 ± 1.3 | 27.0 ± 0.5 | 20.7 ± 1.0 |
|  | *HexNAc(4)Hex(5)Fuc(1)NeuAc(1)* | 3.7 ± 0.1 | 3.2 ± 0.7 | 2.7 ± 0.3 | 3.7 ± 0.5 |
|  | *HexNAc(4)Hex(5)Fuc(2)* | 16.1 ± 1.7 | 11.1 ± 2.1 | 11.4 ± 2.2 | 12.9 ± 2.8 |
|  | *HexNAc(4)Hex(5)Fuc(2)NeuAc(1)* | 1.0 ± 0.1 | 1.0 ± 0.2 | 0.7 ± 0.2 | 0.3 ± 0.1 |
|  | *HexNAc(4)Hex(5)Fuc(3)* | 5.5 ± 0.3 | 2.9 ± 0.8 | 2.8 ± 0.3 | 4.5 ± 0.8 |
|  | *HexNAc(4)Hex(5)Fuc(3)NeuAc(1)* | 0.3 ± 0.0 | 0.2 ± 0.0 | 0.2 ± 0.0 | 0.1 ± 0.0 |
|  | *HexNAc(4)Hex(5)NeuAc(2)* | 0.5 ± 0.1 | 0.4 ± 0.1 | 0.3 ± 0.1 | 0.1 ± 0.0 |
|  | *HexNAc(4)Hex(6)* | 0.2 ± 0.0 | 0.2 ± 0.0 | 0.2 ± 0.0 | 0.3 ± 0.0 |
|  | *HexNAc(4)Hex(6)Fuc(1)* | 0.3 ± 0.1 | 0.4 ± 0.1 | 0.4 ± 0.1 | 0.3 ± 0.1 |
|  | *HexNAc(5)Hex(4)Fuc(1)* | 0.5 ± 0.0 | 0.8 ± 0.2 | 0.7 ± 0.1 | 0.6 ± 0.1 |
|  | *HexNAc(5)Hex(4)Fuc(2)* | 0.5 ± 0.1 | 0.3 ± 0.0 | 0.3 ± 0.0 | 0.3 ± 0.1 |
|  | *HexNAc(5)Hex(4)NeuAc(1)* | 0.1 ± 0.0 | 0.1 ± 0.0 | 0.1 ± 0.0 | 0.1 ± 0.0 |
|  | *HexNAc(5)Hex(5)* | 1.3 ± 0.4 | 1.8 ± 0.3 | 2.1 ± 0.5 | 1.3 ± 0.1 |
|  | *HexNAc(5)Hex(5)Fuc(1)* | 17.9 ± 0.7 | 18.3 ± 0.6 | 20.9 ± 0.7 | 25.4 ± 4.4 |
|  | *HexNAc(5)Hex(5)Fuc(2)* | 7.8 ± 0.9 | 3.8 ± 0.3 | 5.3 ± 0.8 | 7.1 ± 0.2 |
|  | *HexNAc(5)Hex(5)Fuc(3)* | 0.8 ± 0.0 | 0.4 ± 0.1 | 0.5 ± 0.2 | 0.7 ± 0.2 |
|  | *HexNAc(5)Hex(5)NeuAc(1)* | 0.7 ± 0.1 | 0.5 ± 0.1 | 0.1 ± 0.0 | 0.1 ± 0.0 |
|  | *HexNAc(5)Hex(6)* | 0.7 ± 0.1 | 0.5 ± 0.0 | 0.5 ± 0.0 | 0.2 ± 0.0 |
|  | *HexNAc(5)Hex(6)Fuc(1)* | 5.2 ± 0.6 | 8.6 ± 1.2 | 5.7 ± 1.2 | 5.1 ± 1.1 |
|  | *HexNAc(5)Hex(6)Fuc(2)* | 2.0 ± 0.1 | 1.7 ± 0.2 | 1.5 ± 0.2 | 1.3 ± 0.3 |
|  | *HexNAc(5)Hex(6)Fuc(3)* | 0.9 ± 0.2 | 0.3 ± 0.1 | 0.5 ± 0.0 | ND |
|  | *HexNAc(6)Hex(3)Fuc(2)* | 0.3 ± 0.0 | ND | 0.3 ± 0.0 | 0.1 ± 0.0 |
|  | *HexNAc(6)Hex(4)Fuc(1)* | 0.4 ± 0.1 | 0.3 ± 0.1 | 0.4 ± 0.1 | 0.5 ± 0.1 |
|  | *HexNAc(6)Hex(5)* | 1.3 ± 0.4 | 1.1 ± 0.3 | 1.9 ± 0.4 | 1.0 ± 0.0 |
|  | *HexNAc(6)Hex(5)Fuc(1)* | 0.2 ± 0.1 | 0.1 ± 0.0 | 0.2 ± 0.0 | 0.2 ± 0.1 |
|  | *HexNAc(6)Hex(6)* | 0.2 ± 0.0 | 0.1 ± 0.0 | 0.2 ± 0.0 | 0.1 ± 0.0 |
|  | *HexNAc(6)Hex(6)Fuc(1)* | 1.2 ± 0.2 | 1.6 ± 0.3 | 1.5 ± 0.3 | 1.7 ± 0.2 |
|  | *HexNAc(6)Hex(6)Fuc(2)* | 0.4 ± 0.1 | 0.2 ± 0.0 | 0.2 ± 0.0 | 0.3 ± 0.0 |
|  | *HexNAc(6)Hex(7)Fuc(1)* | 0.2 ± 0.0 | 0.3 ± 0.1 | 0.3 ± 0.0 | ND |
| Site occupancy |  | 97.8 ± 0.2 | 97.8 ± 0.2 | 96.7 ± 0.4 | 96.5 ± 0.4 |
|  | | | | | |
| ^a^Glycopeptides were derived from the digestion of HA protein bands by various enzyme (s) as described in the Materials&Methods section to obtain a uniform peptide sequence for individual sites. The glycopeptides containing N38 or N63 were obtained from the sequential digestion by Asp-N and trypsin. N45-peptides were from the digestion by Asp-N and Glu-C. N483-peptides were from trypsin digestion; all other glycopeptides were derived from the digestions by the combination of trypsin and chymotrypsin. The abundance of each glycan at each site was represented as the mean relative abundance of triplicate experiments as well as the standard deviation of the mean. The relative abundance of a glycan at each site for each replicate was calculated using an area ratio of peptides that have a particular glycan over the total peptides at the site.  ^b^Results were obtained from the glycopeptides containing only N45 site (LVQN*SSIGEIC), while glycans in glycopeptides containing the N38 and N45 sites were not accounted for. ND – not determined. | | | | | |
